# Community context and individual factors associated with arrests among young men in a South African township

**DOI:** 10.1371/journal.pone.0209073

**Published:** 2019-01-17

**Authors:** Joan Christodoulou, Lynissa R. Stokes, Jason Bantjes, Mark Tomlinson, Jackie Stewart, Stephan Rabie, Sarah Gordon, Andile Mayekiso, Mary Jane Rotheram-Borus

**Affiliations:** 1 Department of Psychiatry & Biobehavioral Sciences, Semel Institute, University of California, Los Angeles, United States of America; 2 College of Public Health and Human Sciences, Oregon State University, Corvallis, Oregon, United States of America; 3 Department of Psychology, Stellenbosch University, Stellenbosch, South Africa; Geneva University Hospitals, SWITZERLAND

## Abstract

**Background:**

In high-income countries, individual- and community-level factors are associated with increased contact with the criminal justice system. However, little is known about how these factors contribute to the risk of arrest in South Africa, which has one of the highest rates of arrests globally. We examine both individual- and community-level factors associated with arrests among young men living in the townships of Cape Town.

**Methods:**

Data were collected from a stratified community sample of 906 young men aged 18–29 years old living in 18 township neighborhoods. Communities with high and low rates of arrest were identified. Logistic regression models were used to assess which individual-level (such as substance use and mental health status) and community-level (such as infrastructure and presence of bars and gangs) factors predict arrests.

**Results:**

Significant predictors of arrests were substance use, gang activity, being older, more stressed, and less educated. Living in communities with better infrastructure and in more recently established communities populated by recent immigrants was associated with having a history of arrests.

**Conclusions:**

When considering both individual- and community-level factors, substance use and gang violence are the strongest predictors of arrests among young men in South Africa. Unexpectedly, communities with better infrastructure have higher arrest rates. Community programs are needed to combat substance use and gang activity as a pathway out of risk among South African young men.

**Trial registration:**

ClinicalTrials.gov registration #NCT02358226, registered Nov 24, 2014

## Introduction

South Africa (SA) has one of the highest rates of incarceration globally (at 174 per 100,000), most commonly among young men under the age of 35 years [1, 2]. Over 97% of SA’s prison population are men (compared to about 90% in the US) [2, 3]. Violence is pervasive with about half of SA men between the ages of 12 and 22 years old admitting to committing at least one criminal offence, and it is almost always a violent offense [4]. Reports include assaults of others at school (26%), in public places (21.6%) and at home (19.6%), with 92.9% of the victims reporting that they knew the identity of the perpetrator [5]. In addition, 30% of young men report committing physical or sexual violence against their partners [6, 7]. The current study complements previous research focusing on young men’s management of alcohol and drug behaviors as the primary source of criminal acts [8] by considering both individual- and community-level factors associated with ever being arrested in SA.

Substance use is associated with higher rates of township violence [9] and is linked to 75% of homicides, 67% of domestic violence, and 30% of hospital admissions [10]. Gaining access to drugs often involves stealing, selling, or bartering goods and services–each acting as a pathway to engagement in criminal activity. Almost a third of young SA men report symptoms of alcohol dependency; half report recently using cannabis (dagga); and a third recently used methamphetamine (tik) [11–14]. The rates of substance dependence among SA offenders range from 10%–48% for males [15]. In a SA study (N = 1050), 45% of arrestees tested positive for cannabis (dagga), methaqualone (mandrax), opiates, cocaine, amphetamines, and/or benzodiazepines, with cannabis and methaqualone being the most prevalent [8]. Those arrested for housebreaking and drug/alcohol offenses and those arrested at least once before are also more likely to test positive for drugs [8]. Thus, substance use is hypothesized to be associated with ever being arrested among young men living in SA township neighborhoods.

The high rates of substance use, violence, and incarceration in SA have been linked to unemployment, lower incomes, fewer years in school, younger age, being unmarried, and having mental health issues [5, 16]. More recently, attention has been focused on the mental health of individuals who come into contact with the criminal justice system [17, 18]. As with many other countries, SA experiences high rates of neuropsychiatric disorders in its prison system [19, 20]. Thus, we expect these individual factors (less income and education, younger age, and less cohabitation) and mental health risks (more stress and depressed symptoms) to be associated with arrests.

Research often focuses on youth’s background and risk behaviors to understand acts leading to arrests. Yet, globally it has been demonstrated that community disorder, poverty, and infrastructure are linked to rates of crime [21, 22]. Social disorganization theory suggests that where an individual lives has a substantial impact on their propensity to commit crimes or engage in criminal or delinquent behavior [23, 24]. Specifically, in contexts where there are few social bonds, crime and delinquency thrive [23, 24]. However, it is unknown whether these social processes operate in the same way across different disadvantaged neighborhoods [25]. Shaw and McKay’s classic study in Chicago reports an ecological model of crime by mapping thousands of incidents of juvenile delinquency [23]. The results show that high rates of poverty and ethnic heterogeneity, combined with high levels of residential stability and single-parent households, are indicators of high criminality [23, 24]. Further, neighborhoods characterized by poverty and negative familial factors (e.g., neglect) have been found to contribute to high arrest rates among youth in the U.S. [26, 27] and adolescent anti-social acts in Mexico [28]. However, the applicability of social disorganization theory in SA is unclear. The applicability of Western criminological theories to urban SA has been previously demonstrated [29–31], but there are important differences in the ecological dynamics of violent crime across differing cultures [30]. In SA, with the socio-political history of violence and oppression, the socioeconomic conditions of communities are likely to be important contributors to high criminality [29, 31]. However, there are limited data in post-apartheid SA [31].

Research with individuals needs to take into account differences in the properties of the groups (i.e., neighborhoods) in which they belong [32, 33]. Solely individual-based explanations of the causes of public health concerns are insufficient [34, 35]. In some cases, neighborhood-level differences have been found to be significantly associated with outcomes, even when there is no significant variability between neighborhoods. These data suggest that assessing how individual and neighborhood effects are reciprocal and inherently related would lead to a better understanding of neighborhood effects on arrests [32, 34]. However, the relationship between arrests and individual- or community-level risk factors is far less studied in low- and middle-income countries (LMIC), such as SA. In township neighborhoods, infrastructure (formal housing, electricity, access to water, toilets), limited opportunities for employment, gang activity, and the presence of small, informal and often illegal bars (i.e., shebeens) [11] may function to exacerbate or dampen an individual’s risk of being arrested.

The aim of this cross-sectional study is to examine arrest rates across low-resource township neighborhoods in Cape Town, SA. A community sample of young Black-African men was recruited from five areas within two Cape Town townships. Townships are peri-urban settlements that vary significantly in years since establishment, infrastructure, and access to services. In post-apartheid SA, townships experienced a major influx of migrants in search of work, which resulted in the rapid development of new informal township neighborhoods [36]. Accordingly, the older township communities are more formalized with access to municipal roads, a police station, and community clinics. However, the newly established communities are more informal, where the large majority of the residents are living in informal housing (e.g., shacks), with limited access to basic services [37]. Thus, community-level factors such as less formal housing, fewer household sources of water (e.g., toilets), and food security, as well as greater number of bars and violence are hypothesized to be associated with having a history of arrests.

This study examines: (1) the individual- and community-level factors associated with a history of arrests among young men living in the townships of Cape Town, SA; and (2) whether these factors are significant predictors of contact with the criminal justice system. The data used in this article are part of the baseline assessment for a larger longitudinal study investigating the impact of a public health intervention to improve the health of young men living in low-resource communities [38]. Understanding the associations between individual factors, the community context, and having a history of arrests is important to plan interventions which seek to reduce the risk of arrests and re-arrests among young men in SA.

## Methods

The Institutional Review Boards of the University of California, Los Angeles (IRB#14–001587) and Stellenbosch University (N14/08/116) approved this study [38] (https://doi.org/10.1186/s13063-018-2804-3).

### Participants

Community clusters (n = 18) containing approximately 450–600 households, including roughly 50 young men per neighborhood, were identified in two Cape Town townships: Khayelitsha (including Makaza, Harare, Ndlovini) and Mfuleni. In each community, a random spot was selected, and recruitment proceeded house-to-house in concentric circles until a sample of 50 young men aged 18 to 29 was reached. If other household members reported that there was a male that slept there at least four nights a week, the interviewer returned repeatedly to solicit voluntary participation of the young man. Potential participants were only excluded if they were unable to understand the recruiter, appeared to be actively hallucinating, or lacked capacity to consent. Individuals who appeared to be under the influence of alcohol or drugs during the time of recruitment were enrolled in the study, but were only interviewed once they were no longer intoxicated. Assessments were conducted at a local research facility in the township, with transport provided.

Of the 994 men approached, 5% (*n* = 50) declined to participate or were identified but could not be found later to complete the assessment (*n* = 34). The final sample included 906 young men aged 18–29 years old (*M* = 22.2, *SD* = 2.9).

### Assessments

Trained interviewers administered a one-hour interview, entering the young men’s responses on an encrypted mobile phone, which transmits data to an encrypted file at the earliest possible time. Participants were reimbursed with ZAR 70 (about 5 USD) for their time to complete the interview.

### Materials

Each of the following individual-level factors were self-reported by the young men.

#### History of arrests

Participants reported whether they had ever been arrested (1) or not (0). Self-report measures are one of the most common techniques to collect data on delinquent and criminal behavior, and have been shown to demonstrate sufficient reliability and validity [39].

#### Individual-level factors

*Demographics*. Participants reported their age, education level, whether they were living with the family of origin, partnership status (partnered or single), whether they had children, and their employment history. *Risk History*. Reports of recent drug use (alcohol, cannabis, and methamphetamine) were both self-reported and assessed by Rapid Diagnostic Tests (RDT). Alcohol use was measured using the Alcohol Use Disorders Identification Test (AUDIT-C), a brief, reliable, and valid, three-item questionnaire of problematic alcohol use [14, 40, 41]. Participants also self-reported their consumption of alcohol in the last three days as well as cannabis and methamphetamine in the last two days, and were assessed with urine-based RDT sold by CLIAwaived Inc. (San Diego, CA). The cannabis RDT (CLIA-SDDT-10) detects 11-nor-Δ⁹-tetrahydrocannabinol-9-carboxylic acid as low as 50 ng/mL over the last 30 days. The methamphetamine RDT (CLIA-SDDT-16) detects methamphetamine as low as 500 ng/mL over the last four days. Although self-report data has been found to be a valid measure of substance use among this sample, the level of agreement between self-report and RDT can vary between substances [42]. *Health Status*. Participants reported whether they had ever been tested for HIV, whether they had tested for HIV in the last 6 months, their HIV status, and whether they have had any sexually transmitted diseases (STDs). Symptoms of depression were measured using the Center for Epidemiologic Studies of Depression (CES-D) with a cutoff of >16 taken to indicate clinically significant symptoms of depression [43]. The CES-D scale was found reliable in previous research in SA [44]. Perceived stress was also assessed using the Perceived Stress Scale (PSS), a 10-item scale focused on stress over the last month, with a cutoff of >13 to indicate above average stress [45]. The PSS was found valid and reliable in previous studies in Asia [46].

#### Community-level factors

Each of the following community-level factors were measured using: 1) official government areal maps; 2) images from Google Earth, 3) research assistants systematically canvassing the neighborhoods at multiple times during the day and evening on weekdays and weekends, and 4) brief street-intercepted surveys of young men in the neighborhoods (some of whom were recruited for the current study if they lived in the households approached for recruitment and were 18 to 29 years old as previously described). For each of the 18 community clusters, we assessed the level of infrastructure (proportion of formal versus informal housing, source of water (i.e., community pump, water on premises, and whether the household had a flush toilet) and the number of bars (shebeens) by household in each community [11, 47]. Therefore, we mapped the locations of all bars within each community, as government records do not provide a reliable measure of the actual number of these small and informal establishments. This data was consistent with the proportions of infrastructure self-reported by the young men. *Food insecurity* was assessed using one item (“How many days in the past week have you gone hungry?”) from The Household Food Insecurity Access Scale (HFIAS). This item has been found to be highly correlated with the nine-item scale used to distinguish food insecure from food secure households in Cape Town townships and other cultural contexts [48–50]. Violence. Participants self-reported incidences of violent behavior, gang involvement, and violence against women.

### Statistical analyses

First, individual-level and community-level risk factors were compared based on the history of arrests across all neighborhoods. As a first step, chi-squared analyses (*df* = 1) were used to identify significant covariates between young men who have been arrested and those who have not. From these identified covariates, the most relevant variables were entered into a multiple-predictor logistic regression to identify individual- and community- level risk factors associated with arrests. Odds ratios (*OR*) with 95% confidence intervals (*CI*) are presented.

To identify communities with a high versus low number of arrests, we compared the proportion of men who have ever been arrested in each neighborhood. Since less than half of the young men reported ever being arrested, a reasonable split of 25% of the communities with the highest arrest rates (5 neighborhoods, 41.0% young men ever arrested, *n* = 103/251) was compared with 25% of the communities with the lowest arrest rates (5 neighborhoods, 23.8% young men ever arrested, *n* = 60/252). We then compared measures of individual- and community-level risk factors between the communities with a high and low number of arrests using chi-squared analyses (*df* = 1). This enabled analysis between neighborhoods by comparing aggregate measures of community-level risk factors. All analyses were conducted using SPSS Statistics 23 (Chicago, IL) [51].

## Results

All study participants were young Black-African males (*n* = 906, *M*_*age*_ = 22.2, *SD* = 2.9) who had completed on average 10 years of schooling (*M* = 10.3; *SD* = 1.6). One-third of the young men reported having been arrested at least once (33.3%; *n* = 303).

### Individual-level risk factors associated with a history of arrest

Table 1 summarizes the individual-level factors associated with a history of arrest. A history of arrest was associated with: having children (*χ*^*2*^ = 30.62, *p* <.001), never being employed (*χ*^*2*^ = 9.05, *p* = .003), higher self-reported rates of substance use and testing positive for recent substance use on the RDTs (*χ*^*2*^> 7.28, p <.01); and reporting higher rates of depression (*χ*^*2*^ = 8.58, p = .004) and stress (*χ*^*2*^ = 23.28, p <.001). No significant associations were found between ever being arrested and living with the family of origin, partnership status, AUDIT-C scores, or being tested for or living with HIV or other STDs.

**Table 1:** Comparisons of individual-level factors of young men from 18 township neighborhoods in South Africa, based on their history of arrests.

Individual Factors	Arrested (n = 303)	Never Arrested (n = 605)
	Mean (SD)/% (n)	Mean (SD)/% (n)
**Demographics**		
Lives with parents	65% (195/301)	64% (386/605)
Partnership status		
*Girlfriend/wife*	95% (287/301)	94% (570/605)
*Single*	5% (14/301)	6% (35/605)
Has children*	40% (120/301)	22% (135/605)
Employed		
*Employed in the past year*	65.4% (197/301)	59.8% (362/605)
*Highest income ever (ZAR)*	3626 (5257)	3077 (4870)
Never employed*	24% (73/301)	34% (206/605)
**Risk History**		
Alcohol use		
*Self-Report (past 3 days)*	32% (96/301)	29% (177/605)
*Rapid Diagnostic Test (past 78 hours)*	38% (106/283)	28% (164/578)
*AUDIT-C>2*	54% (161/301)	52% (315/605)
Cannabis use*		
*Self-Report (past 2 days)*	48% (143/301)	27% (166/605)
*Rapid Diagnostic Test (past 30 days)*	63% (178/283)	42% (241/578)
Methamphetamine use*		
*Self-Report (past 2 days)*	19% (56/301)	5% (32/605)
*Rapid Diagnostic Test (past 4 days)*	40% (114/283)	14% (78/578)
**Health Status**		
Ever tested for HIV	88% (266/301)	89% (538/605)
Tested HIV (last 6 months)	50% (133/268)	49% (265/538)
Self-reported HIV status		
*Positive*	6% (16/264)	3%(14/533)
*Negative*	83% (220/264)	89% (474/533)
*No results/decline to answer*	11% (28/264)	8% (45/533)
STD ever	8% (23/301)	8% (46/605)
Depressed (CESD score>16)*	41% (122/301)	31% (186/605)
Perceived stress (score>13)*	54% (162/301)	40% (240/605)

*p < .05

### Community-level risk factors associated with a history of arrest

Table 2 summarizes the community-level factors associated with a history of arrest. A history of arrest was associated with: living in formal housing (*χ*^*2*^ = 4.39, *p* <.05); having access to water in the home (*χ*^*2*^ = 6.73, *p* = .011); having a flush toilet in the home (*χ*^*2*^ = 4.96, *p* <.05); and occasions of violence (*χ*^*2*^> 10.38, p <.001). We did not find any significant associations between ever being arrested and having electricity, the number of bars, and food insecurity.

**Table 2:** Comparisons of the community-level factors of young men from 18 township neighborhoods in South Africa, based on their history of arrests.

Community Factors	Arrested (n = 303)	Never Arrested (n = 605)
	Mean (SD)/% (n)	Mean (SD)/% (n)
**Infrastructure**		
Housing type*		
*Formal–brick*	49.8% (150/301)	42.5% (257/605)
*Informal—shack*	50.2% (151/301)	57.5% (348/605)
Water source*		
*In home*	72.4% (218/301)	63.8% (386/605)
*Community tap*	27.6% (83/301)	36.2% (219/605)
Household toilet*		
*Flush toilet*	67.4% (203/301)	59.8% (362/605)
*No Flush toilet*	32.6% (98/301)	40.2% (243/605)
**Food insecurity (past week)**	60.1% (181/301)	54.4% (329/605)
Violence*		
*Violent incident ever*	57.8% (174/301)	46.5% (281/605)
*Gang member*	34.9% (105/301)	13.7% (83/605)
*Hit girlfriend*	46.2% (139/301)	27.9% (169/605)

*p < .05

### Individual- and community-level factors associated with arrests

Ten community and individual factors were significantly associated with a history of arrest in the univariate analysis. These significant factors were entered into a multivariate logistic regression analysis (see Table 3). Having a history of being arrested was associated with testing positive for the following substances: methamphetamine (a*OR* = 3.32, *p* <.001, *95%CI* = 2.20–4.99), cannabis (a*OR* = 1.56, *p* = .014, *95%CI* = 1.10–2.23), and alcohol (a*OR* = 1.65, *p* = .006, *95%CI* = 1.15–2.36). A history of arrest was associated with current gang activity (a*OR* = 2.77, *p* <.001, *95%CI* = 1.85–4.14); being older (a*OR* = 1.19, *p* <.001, *95%CI* = 1.12–1.26), having fewer years of education (a*OR* = 0.86, *p* = .004, *95%CI* = 0.78–0.95), and reporting more stress (a*OR* = 1.26, *p* <.006, *95%CI* = 1.07–1.48). Depression, having a household toilet, and neighborhood were unrelated to a history of arrest (*p’s* >.10).

**Table 3:** Adjusted odds ratios and 95% confidence intervals from multiple logistic regression model assessing individual- and community-level risk factors for being arrested among young men from 18 communities in South Africa.

	*aOR*	95% C.I.	*p* value
Age	1.19	1.12, 1.26	<.001
Education	0.86	0.78, 0.95	.004
Substance Use (RDT)			
Alcohol	1.65	1.15, 2.36	.006
Methamphetamine	3.32	2.20, 4.99	.001
Cannabis	1.56	1.10, 2.23	.014
Perceived stress (score>13)	1.26	1.07, 1.48	.006
Depressed (CESD score>16)	0.91	0.61, 1.35	.627
Household toilet	1.37	0.63, 2.98	.424
Gang Membership	2.77	1.85, 4.14	<.001

Note. Neighborhood (*n* = 18) was also included in this model, *Wald* = 15.34, *df* = 17, *p* = 0.571.

### Comparisons of communities with a high and low number of arrests

**Fig 1:**
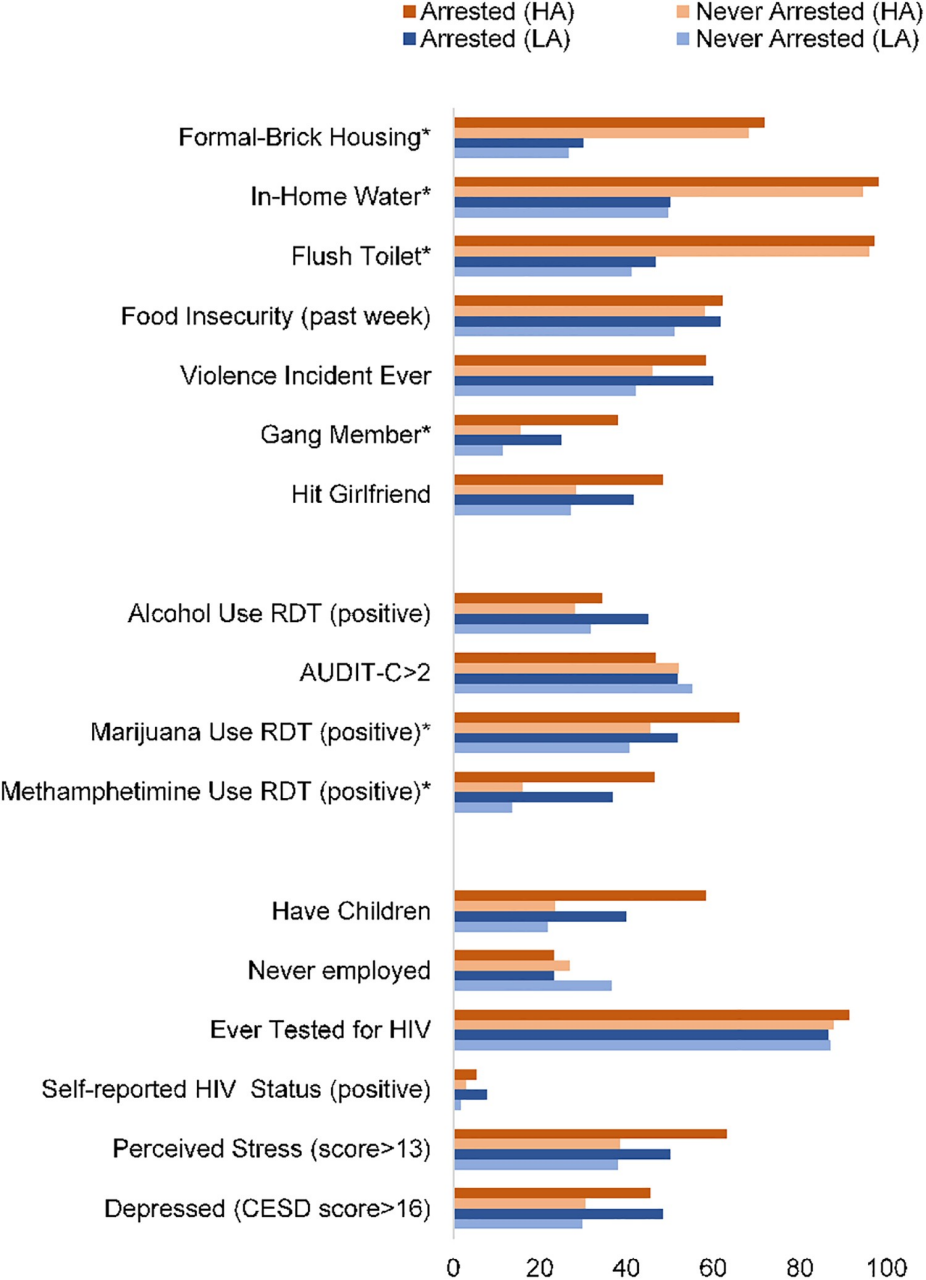
Comparisons of community and individual factors from 5 township communities with the highest (HA; 41.0%, *n* = 103/251) and 5 with lowest rates of arrests (LA; (23.8%, *n* = 60/252).

Among the 18 communities, five were characterized by a high number of arrests (41.0%, *n* = 103/251) and five neighborhoods were characterized by a low number of arrests (23.8%, *n* = 60/252). Fig 1 summarizes the individual- and community-level factors associated with a history of arrest among young men living in these 10 neighborhoods (*n* = 503).

#### Individual-level risk factors

On average, communities with a high number of arrests had a greater proportion of young males testing positive for cannabis (*χ*^*2*^ = 6.77, *p* = .012) and methamphetamine use (*χ*^*2*^ = 6.12, *p* = .015). Neighborhoods with high verses low rates of arrests were unrelated to alcohol use, having children, never being employed, testing for HIV or HIV status, alcohol use, and health status (*p’s* > 0.10).

#### Community-level risk factors

Communities with a high number of arrests were characterized by a greater proportion of formal housing (*χ*^*2*^ = 90.52, *p* <.001), with water inside the home (*χ*^*2*^ = 136.68, *p* <.001) and flush toilets (*χ*^*2*^ = 172.32, *p* <.001), and a higher proportion of males reporting gang activity (*χ*^*2*^ = 7.98, *p* = .005) than communities with a low number of arrests. Neighborhoods with high verses low rates of arrests were unrelated to the number of bars and food insecurity in the communities, (*p’s* > 0.10).

## Discussion

Substance use and gang involvement are the strongest predictors of ever being arrested. Substance use is consistently linked with high levels of violence and gang involvement [4, 6]. Most notably, young men using methamphetamine are three times more likely to be arrested than young men who abstained. Alcohol users are almost twice as likely to report ever being arrested compared to non-drinkers. This is consistent with previous studies in both HIC [52] and LMIC [53, 54]–including SA [8]–that link substance use with arrest history and incarcerations. This is one of the first studies to examine both individual and community characteristics linked to contact with the criminal justice systems among SA men. While poverty is widespread in these peri-urban settlements in Cape Town, this classification of resources may not be sufficient to explain the high rates of criminal activity among different SA communities, especially if more than half of young men report engaging in substance use or risky delinquent acts.

One in five young men report being a gang member in the townships of Cape Town and over half of all young men report committing at least one violent act. These findings are not surprising in these post-apartheid neighborhoods characterized by widespread poverty and limited educational and employment opportunities. Young men who report being involved in a gang are more than twice as likely to report ever being arrested. These findings extend previous research on the widespread culture of violence in the townships [2–6] by examining the association between reports of different types of violence and being arrested in different neighborhoods.

Further, SA young men who report being stressed are more likely to have a history of arrests. Prisoners and arrestees in SA, as with many other countries, experience higher rates of neuropsychiatric disorders than the general population [19, 20]. A large-scale longitudinal analysis in the United States reports that individuals with severe mental illness are only more violent if they also experience substance abuse [18]. Mental health symptoms are rarely studied among men, especially in LMIC, and need more investigation.

Although rates of food insecurity (40%) and unemployment are high in this study, young men living in communities with more formal housing and in-home water sources are more likely to report ever being arrested compared to those in less advantaged communities. Two of the 18 communities with the highest rates of arrests are located in a relatively new township. Prior to any migrants arriving, the government provided paved roads, access to electricity, housing, and in-home water sources. Because this township has the most immigrants, residents are likely to have fewer ties to the community [55]. Although households may have more infrastructure, the lack of long-standing family ties, along with the difficulty in obtaining employment, may contribute to a high risk of arrests. Research in rural areas of the United States suggests that communities characterized by high ethnic heterogeneity and less local engagement among juveniles are consistently linked to higher rates of arrests [56]. Although all of the young men in this study are Black-African, many migrate from the Eastern Cape in SA [36], limiting their bonds to the township communities. These findings support the extension of the social disorganization theory to the township neighborhoods if SA [29–31].

Policing may also be different in township neighborhoods with better infrastructure. Housing and access to municipal services are disproportionally distributed across three police precincts in Khayelitsha and one police precinct in Mfuleni [57–59]. For instance, the precinct in Lingelethu West PP includes a much higher proportion of formal housing (68%), compared to those in Khayelitsha (36%) and Harare (42%) [57]. In contrast, the precinct in Khayelitsha includes 56% informal settlements, compared to 46% in Harare and 25% in Lingelethu West [57]. As certain police precincts include more informal settlements, service delivery and policing become a challenge due to the lack of infrastructure [60]. For the present study, the precincts in Harare covers three of our neighborhoods, of which two are informal settlements. Informal settlements often do not have formal roads and corresponding municipal-issued physical addresses. This may account for the surprisingly higher number of arrests in formal areas in the current study. That is, policing, and subsequent arrests, may be more challenging in informal neighborhoods due to the lack of infrastructure and the police’s ability to detect and detain the accused.

The examination of risk associated with being arrested must consider clusters of individual- and community-level factors that can interact to propagate violence, substance use, and potential criminal behavior. This approach helps inform community interventions to create new pathways for young men. There is some evidence that engaging people in community interventions can be an effective way to address public health problems, such as unwanted pregnancy, substance abuse, violence, and delinquency [61]. However, it is notoriously difficult to engage young men in these types of interventions [38]. As recently mentioned by Brooks (2018), “human behavior happens in contagious, networked ways” [27]. Community-level interventions are needed for community-level challenges in South Africa to combat substance use and gang activity. These findings support the need for pathways out of risk that engage young men in prosocial ways and support their access to education.

### Limitations

This study has several limitations. First, criminal involvement is only measured by self-reports about ever being arrested. The assessment does not have details on the reason for arrests or if the young males were ever convicted and served jail or prison sentences. This cross-sectional study also limits interpretation of the associations. Nonetheless, community uptake was high, and we do appear to have a representative sample of 18 township communities.

### Conclusions

Over 30% of the young men in the current cohort living in the townships of Cape Town, SA have been arrested. These rates are similar to those among African American men in the U.S. (49% by age 23) [62]. The substantial variations in arrest rates across communities suggest that community-level programs are needed to establish pathways out of risk. Consistent with previous research in the U.S. [3], older and less educated men are more likely to report ever being arrested. These findings warrant the need for community programs as part of crime prevention strategies to address social problems, such as widespread substance use and gang activity.

## Supporting information

S1 File S1_Survey Items. English Survey Items and IsiXhosa Survey Items.
(PDF)

S2 File S2_Data. Data.
(XLSX)
