# Supplementary material for: Community context and individual factors associated with arrests among young men in a South African township
Source: PLoS One. 2019 Jan 17;14(1):e0209073. doi: 10.1371/journal.pone.0209073 (PMC6336339; doi:10.1371/journal.pone.0209073)
Supplement: S1 File — English Survey Items and IsiXhosa Survey Items. (PDF) [file pone.0209073.s001.pdf]

ENGLISH SURVEY ITEMS

DOB

What is your date of birth (dd-mm-yyyy)?

Participant education

What is the highest level of education you have completed?
Expects a single option response (required)

- [ ] No schooling [0]
- [ ] Grade 1 / Sub A [1]
- [ ] Grade 2 / Sub B [2]
- [ ] Grade 3 / Std 1 [3]
- [ ] Grade 4/ Std 2 [4]
- [ ] Grade 5 / Std 3 [5]
- [ ] Grade 6 / Std 4 [6]
- [ ] Grade 7 / Std 5 [7]
- [ ] Grade 8 / Std 6 [8]
- [ ] Grade 9 / Std 7 [9]
- [ ] Grade 10 / Std 8 [10]
- [ ] Grade 11/ Std 9 [11]
- [ ] Grade 12/ Matric [12]
- [ ] Post Matric Certificate / Diploma [13]
- [ ] Degree [14]
- [ ] Decline to answer [91]

Participant lives with others

How many people are living under the same roof as you (people who sleep in the household more than 2 nights each week)?

Partner living with you

Is your partner one of these people?

- ☐ Yes [1]
- ☐ No [2]
- ☐ I don't have a current partner [3]

Participant living with parents

Do you live with either of your parents?

- ☐ Yes [1]
- ☐ No [2]

Participant living with their children

Do you live with any of your OWN children?

- ☐ Yes [1]
- ☐ No [2]

Housing description

What best describes your housing?

- ☐ Formal brick structure on a separate yard [1]
- ☐ Informal dwelling / shack in backyard [2]

Water source

What is the main source of drinking water?

- ☐ Water in the home [1]
- ☐ Water on the premises [2]
- ☐ Water from a community tap / public tank [3]

Household toilet

What toilet facilities does your household have?

- [ ] Flush toilet on the premises [1]
- [ ] Public [2]
- [ ] Portable [3]
- [ ] Bucket system [4]
- [ ] Bush [5]

Electricity

Do you have electricity in your household?

- [ ] Yes [1]
- [ ] No [2]

Cooking fuel

What is your main source of fuel for cooking?

- [ ] Electricity [1]
- [ ] Paraffin [2]
- [ ] Gas [3]
- [ ] Coal [4]
- [ ] Wood [5]
- [ ] Other [95]

Work Type

Have you done any of the following types of work?

- ☐ Factory worker [1]
- ☐ Farm worker [2]
- ☐ Sell drugs [3]
- ☐ Vendor [4]
- ☐ Gang member [5]
- ☐ Teacher [6]
- ☐ Driver [7]
- ☐ Shebeen work [8]
- ☐ Admin person [9]
- ☐ Health worker [10]
- ☐ Security [11]
- ☐ Worked in the mines [12]
- ☐ Builder [13]
- ☐ Janitor [14]
- ☐ Sex worker [15]
- ☐ Other [16]
- ☐ Never worked [17]

Participant Days of Hunger

How many days in the passed WEEK have you gone hungry? (By this I mean days when you felt you didn't have enough to eat)

Often Drink Alcohol

How many days in the last two weeks have you had a drink containing alcohol (whether at a shebeen, your home or anywhere else)?

[ ]

Number of Drinks in last 3 days

How many drinks containing alcohol have you had in the last 3 days?

[ ]

Number Drinks to Feel High

How many drinks does it currently take to make you feel high/relaxed?

[ ]

Often Drink 6 or More

In the last three months how often have you had six or more drinks on one occasion?

- ☐ Never [1]
- ☐ Less than monthly [2]
- ☐ Monthly [3]
- ☐ Weekly [4]
- ☐ Daily or Almost daily [5]

Often Find Not Able to Stop Drinking Once Started

How often in the past three montns did you find you were not able to stop drinking once you started?

- ☐ Never [1]
- ☐ Less than monthly [2]
- ☐ Monthly [3]
- ☐ Weekly [4]
- ☐ Daily or Almost daily [5]

Often Need Morning Drink After Heavy Drinking

How often during the past three months did you find you need a drink in the morning to get you going after a heavy drinking session?

- ☐ Never [1]
- ☐ Less than monthly [2]
- ☐ Monthly [3]
- ☐ Weekly [4]
- ☐ Daily or Almost daily [5]

Alcohol Upon Waking

In the past three months have you sometimes taken a drink in the morning when you first get up, even if you haven't had a heavy drinking session?

- ☐ Never [1]
- ☐ Less than monthly [2]
- ☐ Monthly [3]
- ☐ Weekly [4]
- ☐ Daily or Almost daily [5]

Often Failed Doing What Expected Because of Drinking

How often in the past three months have you failed to do what was normally expected from you because of drinking?

- ☐ Never [1]
- ☐ Less than monthly [2]
- ☐ Monthly [3]
- ☐ Weekly [4]
- ☐ Daily or Almost daily [5]

Often Feel Guilt After Drinking

How often in the past three months have you had a feeling of guilt or remorse after drinking?

- ☐ Never [1]
- ☐ Less than monthly [2]
- ☐ Monthly [3]
- ☐ Weekly [4]
- ☐ Daily or Almost daily [5]

Often Unable to Remember What Happened Due to Drinking

How often in the past three months were unable to remember what happened the night before because of your drinking?

- ☐ Never [1]
- ☐ Less than monthly [2]
- ☐ Monthly [3]
- ☐ Weekly [4]
- ☐ Daily or Almost daily [5]

Anyone Injured Due to Drinking

In the last three months have you or someone else been injured as a result of your drinking?

- ☐ Never [1]
- ☐ Less than monthly [2]
- ☐ Monthly [3]
- ☐ Weekly [4]
- ☐ Daily or Almost daily [5]

Anyone Concerned About Your Drinking

Has a friend or relative or doctor or health worker been concerned about your drinking and suggested that you cut down?

- [ ] Yes, in the three months [1]
- [ ] Yes, but not in the last year [2]
- [ ] No [3]

Need to Cut Down Drinking

In the last three months have you sometimes felt the need to cut down on your drinking?

- [ ] Yes [1]
- [ ] No [2]
- [ ] Decline to answer [91]

Memory Loss With Alcohol

In the last three months has a friend or family member ever told you about things you said or did while you were drinking that you could not remember?

- [ ] Yes [1]
- [ ] No [2]
- [ ] Decline to answer [91]

Ever Used Dagga

Have you ever used dagga?

- [ ] Yes [1]
- [ ] No [2]

Last three months dagga

Have you used dagga in the last three months?

- [ ] Yes [1]
- [ ] No [2]

Last two weeks dagga use

Have you used dagga in the last two weeks?

- [ ] Yes [1]
- [ ] No [2]

Last two DAYS dagga use

Have you used dagga in the last two days?

- [ ] Yes [1]
- [ ] No [2]

Ever Used Mandrax

Have you ever used mandrax?

- [ ] Yes [1]
- [ ] No [2]

Last three months mandrax

Have you used mandrax in the last three months?

- [ ] Yes [1]
- [ ] No [2]

Last two weeks mandrax

Have you used mandrax in the last two weeks?

- ☐ Yes [1]
- ☐ No [2]

Last two DAYS mandrax

Have you used mandrax in the last two DAYS?

- ☐ Yes [1]
- ☐ No [2]

Ever Used Tik

Have you ever used tik?

- ☐ Yes [1]
- ☐ No [2]

Last three months Tik use

Have you used Tik in the last three months?

- ☐ Yes [1]
- ☐ No [2]

Last two weeks tik use

Have you used Tik in the last 2 weeks?

- ☐ Yes [1]
- ☐ No [2]

Last two DAYS Tik use

Have you used Tik in the last two DAYS?

- ☐ Yes [1]
- ☐ No [2]

Group violence

Have you ever been part of a group who was attacked?

- ☐ Yes [1]
- ☐ No [2]

Group violence involvement

Have you ever chosen to get involved in a physical fight to support others?

- ☐ Yes [1]
- ☐ No [2]

Physical Fights with Men

How many physical fights have you been in, in your lifetime with other men?

____

Recent Physical Fights with Men

How many physical fights have you been in, in the last 3 months with other men?

____

Physical Fights with Family

How many physical fights have you been in, in your lifetime with your family?

____

Recent Physical Fights with Family

How many physical fights have you been in, in the last 3 months with your family?

Times Arrested

How many times have you been arrested in your life?

Times arrested last 3 months

How many times have you been arrested in the last 3 months?

Prison Sentences

How many times in your life have you been sentenced and put in prison?

Recent Prison Sentences

How many times have you been sentenced and put in prison in the last 3 months?

Gang Member

Have you ever been a member of a gang?

- Yes [1]
- No [2]

Gang member last three months

Have you been a gang member within the last three months?

- ☐ Yes [1]
- ☐ No [2]

Participant Height

Please record the participants height (m):

[ ]

Participant Weight

Please record the participants weight (kg):

[ ]

Clinic visits in the last year

How many times have you been to the clinic in the last year (anchor in time)?

[ ]

Ever Tested TB

Have you ever tested for TB?

- ☐ Yes [1]
- ☐ No [2]
- ☐ Decline to Answer [91]

Age first had sex

How old were you when you had your first sexual partner?

[ ]

Life time female sexual partners

How many female sexual partners have you had in your life?

Expects a numeric response (**required**)

Hit girlfriends

Is this statement TRUE or FALSE: I sometimes hit my girlfriend to keep her in line.

- True [1]
- False [2]

Friends hitting girlfriends

Is this statement TRUE or FALSE: Most of my friends hit their girlfriends to keep them in line.

- True [1]
- False [2]

Often Quarreled With Partner

How many of these women have you hit, pulled, dragged or used a weapon on?

Forced Physical Contact

How many of these women have you forced yourself on, at one time or another?

Threatened to Hurt Partner

In general when you break up with a girlfriend do you threaten to hurt her?

- ☐ Never [1]
- ☐ Once [2]
- ☐ Few [3]
- ☐ Many [4]

Sex with Woman for Presents or Money

Has a woman ever paid you for sex with money or presents?

- ☐ Yes [1]
- ☐ No [2]

Were You The Only Man

Have you ever had sex with a woman while she had sex with more men than you?

- ☐ Yes, other men [1]
- ☐ No, only me [2]

Life time male sexual partners

How many male sexual partners have you had in your life?

[ ]

Recent New Sexual Relationships

Within the past 3 months, have you started any new relationships with a sex partner?

- ☐ Yes [1]
- ☐ No [2]
- ☐ Decline to answer [91]

Female sex partners last 3 months

How many different women have you had sex with in the last three months?

[ ]

Recent Quarrels with Partner

How many of these girlfriends have you hit, pulled, dragged or used a weapon on in the last 3 months?

[ ]

Recent Forced Physical Contact

How many of these women have you forced yourself on in the last 3 months?

[ ]

Recently Threatened to Hurt Partner

In the past 3 months have you threatened to hurt any of these women? Did you do this many times, a few times, once or did it not happen.

- ☐ Never [1]
- ☐ Once [2]
- ☐ Few [3]
- ☐ Many [4]

Would Feel Free to End Relationship

The last time you ended a relationship how did you break it off?

- ☐ Just stopped visiting and having contact [1]
- ☐ Threatened her to leave you alone [2]
- ☐ Sent a sms [3]
- ☐ Met with her to explain that you wanted to end it [4]

Female sex partners last two weeks

How many different women have you had sex with in the last two weeks?

Sexually transmitted diseases

How many times in the last three months have you had a sexually transmitted disease, such as, a skin infection?

Periods when seeing two partners at same time

How many periods in the past three months have you been seeing two different partners at the same time e.g not ending a relationship with one women before starting a new one with another woman?

Tested for HIV ever

How many times in your life have you ever been tested for HIV?

Tested in last 6 months

How many times have you been tested for HIV in the last 6 months?

Depression Scale Prompt

Below are listed ways that describe how you might have felt or behaved during the past week. Please tell me if these statements describe the way you felt or behaved in the past week.

Being Bothered

I was bothered by things that usually don't bother me.

- ☐ None of the time [0]
- ☐ A little of the time [1]
- ☐ Occasionally [2]
- ☐ Most of the time [3]

Poor Appetite

I did not feel like eating: my appetite was poor.

- ☐ None of the time [0]
- ☐ A little of the time [1]
- ☐ Occasionally [2]
- ☐ Most of the time [3]

Feeling the Blues

I felt that I could not shake off the blues even with help from my family and friends.

- ☐ None of the time [0]
- ☐ A little of the time [1]
- ☐ Occasionally [2]
- ☐ Most of the time [3]

Feeling Equal

I felt that I was just as good as other people.

- ☐ None of the time [0]
- ☐ A little of the time [1]
- ☐ Occasionally [2]
- ☐ Most of the time [3]

Lack of Focus

I had trouble keeping my mind on what I was doing.

- ☐ None of the time [0]
- ☐ A little of the time [1]
- ☐ Occasionally [2]
- ☐ Most of the time [3]

Feeling Depressed

I felt depressed.

- ☐ None of the time [0]
- ☐ A little of the time [1]
- ☐ Occasionally [2]
- ☐ Most of the time [3]

Effort

I felt that everything I did was an effort.

- ☐ None of the time [0]
- ☐ A little of the time [1]
- ☐ Occasionally [2]
- ☐ Most of the time [3]

Hope for Future

I felt hopeful about the future.

- ☐ None of the time [0]
- ☐ A little of the time [1]
- ☐ Occasionally [2]
- ☐ Most of the time [3]

Life a Failure

I thought that my life had been a failure.

- ☐ None of the time [0]
- ☐ A little of the time [1]
- ☐ Occasionally [2]
- ☐ Most of the time [3]

Fear

I was fearful.

- ☐ None of the time [0]
- ☐ A little of the time [1]
- ☐ Occasionally [2]
- ☐ Most of the time [3]

Restless Sleep

My sleep was restless.

- ☐ None of the time [0]
- ☐ A little of the time [1]
- ☐ Occasionally [2]
- ☐ Most of the time [3]

Happy

I was happy.

- ☐ None of the time [0]
- ☐ A little of the time [1]
- ☐ Occasionally [2]
- ☐ Most of the time [3]

Quieter Than Usual

I talked less than usual.

- ☐ None of the time [0]
- ☐ A little of the time [1]
- ☐ Occasionally [2]
- ☐ Most of the time [3]

Lonely

I felt lonely.

- ☐ None of the time [0]
- ☐ A little of the time [1]
- ☐ Occasionally [2]
- ☐ Most of the time [3]

Unfriendly People

I felt that people were unfriendly.

- ☐ None of the time [0]
- ☐ A little of the time [1]
- ☐ Occasionally [2]
- ☐ Most of the time [3]

Enjoyment of Life

I enjoyed life.

- ☐ None of the time [0]
- ☐ A little of the time [1]
- ☐ Occasionally [2]
- ☐ Most of the time [3]

Crying

I had crying spells.

- ☐ None of the time [0]
- ☐ A little of the time [1]
- ☐ Occasionally [2]
- ☐ Most of the time [3]

Sadness

I felt sad.

- ☐ None of the time [0]
- ☐ A little of the time [1]
- ☐ Occasionally [2]
- ☐ Most of the time [3]

People Dislike Me

I felt that people dislike me.

- ☐ None of the time [0]
- ☐ A little of the time [1]
- ☐ Occasionally [2]
- ☐ Most of the time [3]

Motivation

I could not get 'going'

- ☐ None of the time [0]
- ☐ A little of the time [1]
- ☐ Occasionally [2]
- ☐ Most of the time [3]

Perceived Stress Scale Prompt

The questions in this scale ask you about your feelings and thoughts during the last month. In each case, please indicate how often you felt or thought a certain way.

Unexpectedly Upset

In the last month, how often have you been upset because of something that happened unexpectedly?

- ☐ Never [0]
- ☐ Almost never [1]
- ☐ Sometimes [2]
- ☐ Fairly often [3]
- ☐ Very often [4]

Unable to Control

In the last month, how often have you felt that you were unable to control important things in your life?

- ☐ Never [0]
- ☐ Almost never [1]
- ☐ Sometimes [2]
- ☐ Fairly often [3]
- ☐ Very often [4]

Nervous and Stressed

In the last month, how often have you felt nervous and 'stressed'?

- ☐ Never [0]
- ☐ Almost never [1]
- ☐ Sometimes [2]
- ☐ Fairly often [3]
- ☐ Very often [4]

Confident in Personal Problems

In the last month, how often have you felt confident about your ability to handle personal problems?

- ☐ Never [0]
- ☐ Almost never [1]
- ☐ Sometimes [2]
- ☐ Fairly often [3]
- ☐ Very often [4]

Things Going Your Way

In the last month, how often have you felt that things were going your way?

- ☐ Never [0]
- ☐ Almost never [1]
- ☐ Sometimes [2]
- ☐ Fairly often [3]
- ☐ Very often [4]

Not Coping

In the last month, how often have you found that you could not cope with all the things that you had to do?

- ☐ Never [0]
- ☐ Almost never [1]
- ☐ Sometimes [2]
- ☐ Fairly often [3]
- ☐ Very often [4]

Controlling Irritations

In the last month, how often have you been able to control your irritations in life?

- [ ] Never [0]
- [ ] Almost never [1]
- [ ] Sometimes [2]
- [ ] Fairly often [3]
- [ ] Very often [4]

On Top of Things

In the last month, how often have you felt that you were on top of things?

- [ ] Never [0]
- [ ] Almost never [1]
- [ ] Sometimes [2]
- [ ] Fairly often [3]
- [ ] Very often [4]

Felt Anger

In the last month, how often have you been angered because of things that were outside of your control?

- [ ] Never [0]
- [ ] Almost never [1]
- [ ] Sometimes [2]
- [ ] Fairly often [3]
- [ ] Very often [4]

Overcoming Difficulties

In the last month, how often have you felt difficulties were piling up so high that you could not overcome them?

- ☐ Never [0]
- ☐ Almost never [1]
- ☐ Sometimes [2]
- ☐ Fairly often [3]
- ☐ Very often [4]

ISIXHOSA SURVEY ITEMS

DOB

Yintoni umhla wokuzalwa (dd-mm-yyyy)?

Participant education

Ufunde waphela kweliphi ibanga esikolweni?

- [ ] Ayifundo [0]
- [ ] Grade 1 / Sub A [1]
- [ ] Grade 2 / Sub B [2]
- [ ] Grade 3 / Std 1 [3]
- [ ] Grade 4/ Std 2 [4]
- [ ] Grade 5 / Std 3 [5]
- [ ] Grade 6 / Std 4 [6]
- [ ] Grade 7 / Std 5 [7]
- [ ] Grade 8 / Std 6 [8]
- [ ] Grade 9 / Std 7 [9]
- [ ] Grade 10 / Std 8 [10]
- [ ] Grade 11/ Std 9 [11]
- [ ] Grade 12/ Matric [12]
- [ ] Post Matric Certificate / Diploma [13]
- [ ] Degree [14]
- [ ] Decline to answer [91]

Participant lives with others

Bangaphi abantu ohlala nabo kulendlu uhlala kuyo (abantu abalal apho ngaphezu kwentsuku ezimbini kwiveki nganye)?

☐

Partner living with you
Ingaba iqabane lakho lingomnye waba bantu?

☐ Ewe [1]
☐ Hayi [2]
☐ Andinalo iqabane ngalomzuzu [3]

Participant living with parents

Uhlala nomye wabazali wakho?

☐ Ewe [1]
☐ Hayi [2]

Participant living with their children

Ukhona umntana wakho ohlala naye?

☐ Ewe [1]
☐ Hayi [2]

Housing description
Yeyiphi eyona izoba indlu yakho?

☐ yindlu yesitena kwisiza esizimeleyo. [1]
☐ Lityotyombe ilisemva kwendlu. [2]

Water source

Amanzi eniwaselayo niwafumana kowuphi umthombo?

Expects a single option response (**required**)

- ☐ Amanzi alapha endlini [1]
- ☐ Amanzi akhoyo apha esizeni [2]
- ☐ Amanzi afumaneka kwimpompi kawonke wonke [3]

Household toilet

Nisebenzisa ziphi intlobo zezindlu zangasese?

Expects a single option response (**required**)

- ☐ Indlu yangasese elapha esizeni egungxulwayo [1]
- ☐ Indlu yangasese ka wonke-wonke [2]
- ☐ Potapota [3]
- ☐ Indlu yangasese esebenzisa amabhakethi [4]
- ☐ Endle/ematyholweni [5]

7.5

Electricity

Ninawo umbane ekhayeni lakho?

Expects a single option response (**required**)

- ☐ Ewe [1]
- ☐ Hayi [2]

7.6

Cooking fuel

Nisebenzisa yiphi indlela yokupheka?

Expects a single option response (**required**)

- ☐ Umbane [1]
- ☐ Iparaffini [2]
- ☐ Irhasi [3]

- [ ] Amalahle [4]
- [ ] Iinkuni [5]
- [ ] Okunye [95]

Work Type

Ukhe wenza enye kwezi ntlobo zilandelayo zomsebenzi?

Expects multiple selected options (**required**)

- [ ] Umsebenzi wase Factory [1]
- [ ] Umsebenzi wase fama [2]
- [ ] Wathengisa iziyobisi [3]
- [ ] Wathengisa esitalatweni [4]
- [ ] Ilungu lemigulugudu [5]
- [ ] Utishala [6]
- [ ] Umqhubi [7]
- [ ] Umsebenzi esimokolweni [8]
- [ ] Translate from English to Xhosa: Admin person [9]
- [ ] Unompilo [10]
- [ ] Unogada [11]
- [ ] Umsebenzi wase zimine [12]
- [ ] Umakhi [13]
- [ ] Translate from English to Xhosa: Janitor [14]
- [ ] Wathegisa ngomzimba [15]
- [ ] Okunye [16]
- [ ] Zange uphangele [17]

Participant Days of Hunger

Zingaphi intsuku kule veki iphelileyoobulambileyo ngazo (ndithetha ngeentsuku apho uziqondileyo ukuba ubufuna okunye ukutya)

Often Drink Alcohol

Kukangaphi usela isiselo esineqondo lotywala kwezi veki zimbini zidlulileyo.(nokuba kuse simokolweni,ekhaya okanye noba kuphi na)?

☐

Number of Drinks in last 3 days

Zingaphi iziselo ezineqondo lotywala okziselileyo kwezi ntsuku zintathu zidlulileyo?

☐

Number Drinks to Feel High

Kuthatha iziselo ezingaphi ngoku ukuze uzive unxilile?

☐

Often Drink 6 or More

Kwezinyanga zintathu zidlulileyo kukangaphi usela iziselo ezintandathu nangaphezulu ngaxesha linye?

- ☐ Zange [1]
- ☐ Ngaphantsi kwenyanga nganye [2]
- ☐ Roqo ngenyanga [3]
- ☐ Roqo ngeveki [4]
- ☐ Roqo ngosuku okanye phantse ntsuku zonke [5]

Often Find Not Able to Stop Drinking Once Started

Kukangaphi kwezi veki zintathu zidlulileyo ufumanisa ukuba awukwazi ukuyeka ukusela xa uthe waqalisa?

Expects a single option response (**required**)

- ☐ Zange [1]
- ☐ Ngapantsi kwenyanga [2]
- ☐ Roqo ngenyaka [3]
- ☐ Roqo ngeveki [4]
- ☐ Roqo ngosuku okanye phantse ntsuku zonke [5]

11.10

Often Need Morning Drink After Heavy Drinking

Kukangaphi kwezi nyanga zintathu zidlulileyo ufumanisa ukuba ufuna isiselo kwakusasa ukuze izive usemandleni emva kokusela kakhulu.

Expects a single option response (**required**)

- ☐ Zange [1]
- ☐ Ngaphantsi kwenyanga [2]
- ☐ Roqo ngenyanga [3]
- ☐ Roqo ngeveki [4]
- ☐ Roqo ngosuku okanye phantse ntsuku zonke [5]

11.11

Alcohol Upon Waking

Kwezinyanga zintathu zidlulileyo ukhe wasela isiselo kwakusasa ngamanye amaxesha uvuka nje,noba khange usele kakhulu ngaphambili?

Expects a single option response (**required**)

- ☐ Zange [1]
- ☐ Ngaphantsi kwenyanga [2]
- ☐ Roqo ngenyanga [3]
- ☐ Roqo ngeveki [4]
- ☐ Roqo ngemini okanye phantse ntsuku zonke [5]

11.12

Often Failed Doing What Expected Because of Drinking

Kwezi nyanga zintathu zidlulileyo kukangaphi usohlulwa kukwenza oko bekulindelikileyo kuwe ngenxa yoba ubusele?

Expects a single option response (**required**)

- Zange [1]
- Ngaphantsi kwenyanga [2]
- Roqo ngenyanga [3]
- Rhoqo ngeveki [4]
- Rhoqo ngosuku okanye phantse ntsuku zonke [5]

11.13

Often Feel Guilt After Drinking

Kwezinyanga zintathu zidlulileyo kukangaphi uziva unokuzisola emva kokusela?

Expects a single option response (**required**)

- Zange [1]
- Ngaphantsi kwenyanga [2]
- Rhoqo ngenyanga [3]
- Rhoqo ngeveki [4]
- Ntsuku zonke okanye phantse ntsuku zonke [5]

11.14

Often Unable to Remember What Happened Due to Drinking

Kwezi nyanga zintathu zidlulileyo kukangaphi ungakwazi ukukhumbula okwenzekileyo kubusuku obudlulileyo ngenxa yoba ubusele?

Expects a single option response (**required**)

- Zange [1]
- Ngaphantsi kwenyanga [2]
- Nyanga ne nenyanga [3]
- Veki neveki [4]
- Ntsuku zonke okanye phantse ntsuku zonke [5]

11.15

Anyone Injured Due to Drinking

Kwezi nyanga zintathu zidlulileyo ingaba wena okanye omnye umntu ukhe wonzakala na ngenxa yokusela kwakho?

Expects a single option response (**required**)

- ☐ Zange [1]
- ☐ Ngaphantsi kwenyaka [2]
- ☐ Nyanga nenyanga [3]
- ☐ Veki ne veki [4]
- ☐ Ntsuku zonke okanye phantse ntsuku zonke [5]

11.16

Anyone Concerned About Your Drinking

Kwezi nyanga zintathu zidlulileyo umhlobo, uGqirha okanye uNompilo ukhe wanexhala na ngokusela kwakho wade wacebisa ukuba wehlise ukusela?

Expects a single option response (**required**)

- ☐ Ewe kulo nyaka udlulileyo [1]
- ☐ Ewe, kodwa akukho kulonyaka udlulileyo [2]
- ☐ Hayi [3]

11.17

Need to Cut Down Drinking

Kwezi nyanga zintathu zidlulileyo ukhe kwakho ixesha lokuba uqonde ukuba kumele usele kancinci?

Expects a single option response (**required**)

- ☐ Ewe [1]
- ☐ Hayi [2]
- ☐ Ndiyala ukuphendula [91]

11.18

Memory Loss With Alcohol

Kwezi nyanga zintathu zidlulileyo ingaba umhlobo okanye ilungu losapho likhe lakuxelela na ngezinto ozenzileyo okanye ozithethileyo ngexesha ubusele nobungako wena ukuzikhumbula?

Expects a single option response (**required**)

- Ewe [1]
- Hayi [2]
- Ndiyala ukuphendula [91]

Ever Used Dagga

Wakhe wayitshaya intsangu?

Expects a single option response (**required**)

- Ewe [1]
- Hayi [2]

Branches

If response **Equals 'Hayi [2]'** then skip to ***Ever Used Mandrax (11.24)***

11.21

Last three months dagga

Ukhe watshaya intsangu kwinyanga ezintathu ezidlulileyo?

Expects a single option response (**required**)

- Ewe [1]
- Hayi [2]

Branches

If response **Equals 'Hayi [2]'** then skip to ***Ever Used Mandrax (11.24)***

11.22

Last two weeks dagga use

Ukhe watshaya intsangu kwezi veki zimbini zidlulileyo?

Expects a single option response (**required**)

- Ewe [1]
- Hayi [2]

11.23

Last two DAYS dagga use

Ukhe watshaya intsangu kwezi ntsuku zimbini zidlulileyo?

Expects a single option response (**required**)

- Ewe [1]
- Hayi [2]

11.24

Ever Used Mandrax

Wakhe watshaya iMandrax?

Expects a single option response (**required**)

- Ewe [1]
- Hayi [2]

Branches

If response **Equals 'Hayi [2]'** then skip to ***Ever Used Tik (11.28)***

11.25

Last three months mandrax

Ukhe watshaya iMandrax kwezi nyanga zintatthu zidlulileyo ?

Expects a single option response (**required**)

- Ewe [1]
- Hayi [2]

Branches

If response **Equals 'Hayi [2]'** then skip to ***Ever Used Tik (11.28)***

11.26

Last two weeks mandrax

Ukhe watshaya iMandrax kwezi veki zimbini zidlulileyo?

Expects a single option response (**required**)

- Ewe [1]
- Hayi [2]

11.27

Last two DAYS mandrax

Ukhe watshaya iMandrax kwezi ntsuku zimbini zidlulileyo?

Expects a single option response (**required**)

- Ewe [1]
- Hayi [2]

11.28

Ever Used Tik

Wakhe watshaya iTik?

Expects a single option response (**required**)

- Ewe [1]
- Hayi [2]

Branches

If response **Equals 'Hayi [2]'** then skip to ***Girlfriends Instruction (12.1)***

11.29

Last three months Tik use

Ukhe watshaya iTik kwezi nyanga zintathu zidlulileyo?

Expects a single option response (**required**)

- Ewe [1]
- Hayi [2]

Branches

If response **Equals 'Hayi [2]'** then skip to ***Girlfriends Instruction (12.1)***

11.30

Last two weeks tik use

Ukhe watshaya iTik kwezi veki zimbini zidlulileyo?

Expects a single option response (**required**)

- Ewe [1]
- Hayi [2]

11.31

Last two DAYS Tik use

Ukhe watshaya iTik kwezi ntsuku zimbini zidlulileyo?

Expects a single option response (**required**)

- [ ] Ewe [1]
- [ ] Hayi [2]

Group violence

Ukhe waba yinxalenye yeqela elakhe lahlaselwa?

Expects a single option response (**required**)

- [ ] Ewe [1]
- [ ] Hayi [2]

15.2

Group violence involvement

Ukhe wakhetha ukuba yinxalenye yomlo ubonisa inkxaso kwabanye?

Expects a single option response (**required**)

- [ ] Ewe [1]
- [ ] Hayi [2]

15.3

Physical Fights with Men

Mingaphi imilo okhe waba kuyo apha ebomini bakho namanye amadoda?

Expects a numeric response (**required**)

Branches

If response **Equals '0'** then skip to ***Physical Fights with Family (15.5)***

15.4

Recent Physical Fights with Men

Mingaphi imilo okhe waba kuyo namanye amadoda kwinyanga ezintathu ezidlulileyo?

Expects a numeric response (**required**)

15.5

Physical Fights with Family

Mingaphi imilo ebe kuyo namalungu osapho lwakho apha ebomini bakho?

Expects a numeric response (**required**)

Branches

If response **Equals '0'** then skip to ***Times Arrested (15.7)***

15.6

Recent Physical Fights with Family

Mingaphi imilo obekuyo namalungu osapho kwinyanga ezintathu ezidlulileyo?

Expects a numeric response (**required**)

15.7

Times Arrested

Kungaphi apha ebomini bakho ubanjwa?

Expects a numeric response (**required**)

Branches

If response **Equals '0'** then skip to ***Sex with Man for Presents or Money (15.11)***

15.8

Times arrested last 3 months

Kungaphi ubanjwa kwezinyanga zintathu zidlilileyo?

Expects a numeric response (**required**)

15.9

Prison Sentences

Kungaphi ubanjwa ubwetywe de uthunyelwe entolongweni?

Expects a numeric response (**required**)

Prerequisites
Skip when ***Prison Sentences (15.9)*** **Equals '0'**

15.10

Recent Prison Sentences

Kukangaphi ugwetywa de uthunyelwe entolongweni kwezi nyanga zintathu zidlulileyo?

Expects a numeric response (**required**)

15.11

15.13

Gang Member

Wakhe waba lilungu loo nqalintloko?

Expects a single option response (**required**)

- [ ] Ewe [1]
- [ ] Hayi [2]

Prerequisites
Skip when ***Gang Member (15.13)*** **Equals 'Hayi [2]'**

15.14

Gang member last three months

ingaba ukhe walilungu lonqalintloko kwezi nyanga zintathu zidlulileyo?

Expects a single option response (**required**)

- [ ] Ewe [1]
- [ ] Hayi [2]

Participant Height

Nceda ufake ubude bomthathi-nxaxheba(m):

Participant Weight

Nceda ufake ubunzima bomthathi-nxaxheba(kg):

Clinic visits in the last year

Kukangaphi usiya eclinic kulonyaka uphelileyo(anchor in time)?

Ever Tested TB

Wakhe wahlolelwa isifo sephepha?

- ☐ Ewe [1]
- ☐ Hayi [2]
- ☐ Ndiyala ukuphendula [91]

Age first had sex

Wawungakanani xa wawuqala ukuba neqabane lakho lezesondo?

Expects a numeric response (**required**)

14.2

Life time female sexual partners

Mangaphi amaqabane akho ezesondo obenawo apha ebemini?

Expects a numeric response (**required**)

14.3

Hit girlfriends

Ingaba oku kuyinyani okanye akuyonyani: Ngamanye amaxesha ndikhe ndilibethe iqabane lam ngeenjongo zokumoluleka.

Expects a single option response (**required**)

- ☐ Yinyani [1]
- ☐ Ayonyani [2]

14.4

Friends hitting girlfriends

Ingaba oku kuyinyani okanye akuyonyani:Uninzi lwabahlobo bam bayawabetha amaqabane abo ngeenjongo zokubaluleka

Expects a single option response (**required**)

- ☐ Yinyani [1]
- ☐ Akuyonyani [2]

14.5

Often Quarreled With Partner

Kwabo basetyhini bangaphi oye wababetha,wabatsala, wabarhuqa okanye wasebenzisa isikhali kubo?

Expects a numeric response (**required**)

14.6

Forced Physical Contact

Kula mantombazana mangaphi okhe wazinyanzela kuwo, kwakanye okanye ngaphezulu.

Expects a numeric response (**required**)

14.7

Threatened to Hurt Partner

Ngokuqhelekileyo xa usohlukana neqabane lakho uye uligrogrise ngokulonzakalisa?

Expects a single option response (**required**)

- Zange [1]
- Kanye [2]
- Kambalwa [3]
- Kaninzi [4]

14.8

Sex with Woman for Presents or Money

Ingaba ukhona owasetyhini owakhe wakubhatala ngemali okanye ngesipho ukuze ulale naye?

Expects a single option response (**required**)

- Ewe [1]
- Hayi [2]

14.9

Were You The Only Man

Wakhe wabelana na ngesondo nomntu wasetyhini, xeshanye namanye amadoda ngaphandle kwakho?

Expects a single option response (**required**)

- Ewe namanye amadoda [1]
- Hayi mna ndodwa [2]

14.10

Life time male sexual partners

Mangaphi amadoda owabelene nawe ngesondo apha ebomini bakho?

Expects a numeric response (**required**)

14.11

Recent New Sexual Relationships

Kwezi nyanga zintathu zidlulileyo ukhe waqala ubudlelwane obutsha neqabane lezesondo?

Expects a single option response (**required**)

- Ewe [1]
- Hayi [2]
- Ndiyala ukuphendula [91]

14.12

Female sex partners last 3 months

Bangaphi abasetyhini owabelene nabo ngesondo kwezi nyanga zintath zidlulileyo?

Expects a numeric response (**required**)

14.13

Recent Sex with Woman for Presents or Money

Ingaba ukhona owasetyhini okhe wakubhatala ngemali okanye isipho kuba uleli naye?

Expects a single option response (**required**)

- Ewe [1]
- Hayi [2]

14.14

Recent Quarrels with Partner

Bangaphi kwaba basetyhini obabethileyo,wabatsala, wabarhuqa okanye wasebenzisa isikhali kubo kwezi nyanga zintathu zidlulileyo?

Expects a numeric response (**required**)

14.15

Recent Forced Physical Contact

Mangaphi kulamanenekaze owazinyanzelayo kuye kwenyanga zintathu zidlulileyo?

Expects a numeric response (**required**)

14.16

Recently Threatened to Hurt Partner

Kwezinyanga zintathu zidlulileyo ukhe wagrogrisa omnye waba basetyhini ? Ukwenza oku amaxesha amaninzi, amaxesha ambalwa, kanye okanye zange kwenzeke?

Expects a single option response (**required**)

- Zange [1]
- Kanye [2]
- Amaxesha ambalwa [3]
- Amaxesha amaninzi [4]

14.17

Would Feel Free to End Relationship

Kwityeli lokugqibela usohlukana neqabane lakho, niyiphelise njani/iphele njani?

Expects a single option response (**required**)

- Wasuka wayeka ukumndwendwela nokuxhumana naye [1]
- Wamgrogrisa ukuba makohlukane nawe [2]
- Wamthumelela umyalezo ngomnxeba [3]
- Nadibana wamcacisela ukuba ufuna ukuyiphelisa [4]

14.18

Female sex partners last two weeks

Bangaphi abasetyhini oleli nabo kweziveki zimbini zidlulileyo

Expects a numeric response (**required**)

14.19

Sexually transmitted diseases

Kukangaphi kwezi nyanga zidlulileyo usiba nezifo ezosulela ngezesondo, ezifana nosuleleko lwesikhumba?

Expects a numeric response (**required**)

14.20

Periods when seeing two partners at same time

Mangaphi amatyeli kwisithuba seenyanga ezintathu othe waba nabaqabane amabini ohlukeneyo ngexesha elinye, umzekelo, ungohlukananga nomnye uqale enye nomnye?

Expects a numeric response (**required**)

14.21

Tested for HIV ever

Uhlolwe kangambi isandulela ngculaza apha ebomini bakho

Expects a numeric response (**required**)

Branches

If response **Equals '0'** then skip to ***Shared Status with Partners (14.28)***

14.22

Tested in last 6 months

Uhlolwe kangaphi isandulela ngculaza kwezi nyanga zintandathu zidlulileyo?

Depression Scale Prompt

Ngezantsi kukho uluhlu lwezinto ezichaza indlela onokuba ubuva ngayo okanye uziphethe ngayo kule veki idlulileyo. Nceda undixelele ukuba ingaba oku kuchaza lendlela ubusiva okanye uziphethe ngayo kuleveki iphelileyo.

18.2

Being Bothered

Bendikhathazekile zizinto ebezingaqhelanga kundikhathaza.

Expects a single option response (**required**)

- ☐ Nakanye [0]
- ☐ Ixheshana elincinci [1]
- ☐ Ngamaxesha athile [2]
- ☐ Kwixesha elininzi [3]

18.3

Poor Appetite

Khange ndizive ndifuna ukutya, umdla wam ekutyeni ubumncinci kakhulu.

Expects a single option response (**required**)

- ☐ Nakanye [0]
- ☐ Ixesha elincinci [1]
- ☐ Ngamaxesha athile [2]
- ☐ Ixesha elininzi [3]

18.4

Feeling the Blues

Translate from English to Xhosa: I felt that I could not shake off the blues even with help from my family and friends.

Expects a single option response (**required**)

- ☐ Translate from English to Xhosa: None of the time [0]
- ☐ Translate from English to Xhosa: A little of the time [1]
- ☐ Translate from English to Xhosa: Occasionally [2]
- ☐ Translate from English to Xhosa: Most of the time [3]

18.5

Feeling Equal

Ndizive ndisemandleni ngokufanayo nje nabanye abantu.

Expects a single option response (**required**)

- ☐ Nakanye [0]
- ☐ Ixesha elincinci [1]
- ☐ Ngamaxesha athile [2]
- ☐ Ixesha elininzi [3]

18.6

Lack of Focus

Ndizive kunzima ukuba ndizinzise ingqondo yam kwinto endiyenzayo.

Expects a single option response (**required**)

- ☐ Nakanye [0]
- ☐ Ixesha elincinci [1]
- ☐ Ngamaxesha athile [2]
- ☐ Ixesha elininzi [3]

18.7

Feeling Depressed

Ndizive ndiphantsi koxinzelelo.

Expects a single option response (**required**)

- ☐ Nakanye [0]
- ☐ Ixesha elincinci [1]
- ☐ Ngamaxesha athile [2]
- ☐ Ixesha elininzi [3]

18.8

Effort

Ndaziva ukuba yonke into endiyenzayo ingumzamo.

Expects a single option response (**required**)

- ☐ Nakanye [0]

- [ ] Ixesha elincinci [1]
- [ ] Ngamaxesha athile [2]
- [ ] Ixesha elininzi [3]

18.9

Hope for Future

Ndizive ndinethemba ngekamva.

Expects a single option response (**required**)

- [ ] Nakanye [0]
- [ ] Ixesha elincinci [1]
- [ ] Ngamaxesha athile [2]
- [ ] Ixesha elininzi [3]

18.10

Life a Failure

Bendicinga ukuba ubomi bam buphalele.

Expects a single option response (**required**)

- [ ] Nakanye [0]
- [ ] Ixesha elincinci [1]
- [ ] Ngamaxesha athile [2]
- [ ] Ngamaxesha amanini [3]

18.11

Fear

Bendisoyika.

Expects a single option response (**required**)

- [ ] Translate from English to Xhosa: None of the time [0]
- [ ] Ixeshana elincinci [1]
- [ ] Ngamaxesha athile [2]
- [ ] ixesha elininzi [3]

18.12

Restless Sleep

Bekunzima ukulala.

Expects a single option response (**required**)

- Translate from English to Xhosa: None of the time [0]
- Ixesha elincinci [1]
- Ngamaxesha athile [2]
- Ixesha elininzi [3]

18.13

Happy

Bendonwabile.

Expects a single option response (**required**)

- Khange kwenzeke [0]
- Ixesha elincinci [1]
- Ngamaxesha athile [2]
- Ixesha elininzi [3]

18.14

Quieter Than Usual

Bendithetha kancinci kunesqhelo.

Expects a single option response (**required**)

- Khange kwenzeke [0]
- Ixesha elincinci [1]
- Ngamaxesha athile [2]
- Ixesha elininzi [3]

18.15

Lonely

Ndizive ndililolo.

Expects a single option response (**required**)

- Khange kwenzeke [0]
- Ixesha elincinci [1]

- [ ] Ngamaxesha athile [2]
- [ ] Ixesha elininzi [3]

18.16

Unfriendly People

Bendisiva ngathi abantu abanabubele.

Expects a single option response (**required**)

- [ ] Khange kwenzeke [0]
- [ ] Ixesha elincinci [1]
- [ ] Ngamaxesha athile [2]
- [ ] Ixesha elithile [3]

18.17

Enjoyment of Life

Bendibonwabele ubomi bam.

Expects a single option response (**required**)

- [ ] Nakanye [0]
- [ ] Ixesha elincinci [1]
- [ ] Ngamaxesha athile [2]
- [ ] Ixesha elininzi [3]

18.18

Crying

Bendilila msinya.

Expects a single option response (**required**)

- [ ] Khange kwenzeke [0]
- [ ] Ixesha elincinci [1]
- [ ] Amaxesha athile [2]
- [ ] Ixesha elininzi [3]

18.19

Sadness

Bendiziva ndikhathazekile

Expects a single option response (**required**)

- Khange kwenzeke [0]
- Ixesha elincinci [1]
- Ngamaxesha athile [2]
- Ixesha elininzi [3]

18.20

People Dislike Me

Ndizive ngathi abantu abandithandi.

Expects a single option response (**required**)

- Khange kwenzeke [0]
- Ixesha elincinci [1]
- Ngamaxesha athile [2]
- Amaxesha amaninzi [3]

18.21

Motivation

Bendingakwazi ukuqhubela phambili.

Expects a single option response (**required**)

- Khange kwenzeke [0]
- Ixesha elincinci [1]
- Ngamaxesha athile [2]
- Ixesha elininzi [3]

Perceived Stress Scale Prompt

Imibuzo kwesi sikali imalunga neemvakalelo kunye neengcinga zakho kule nyanga idlulileyo, kumba ngamnya nceda ubonise ukuba kukangakanani usiva okanye ucinga ngohlobo oluthile.

20.2

Unexpectedly Upset

Kulenyanga iphelileyo kukangaphi usiba nomsindo kuba kukho into eyenzekileyo ngokungalindelekanga?

Expects a single option response (**required**)

- ☐ Zange [0]
- ☐ mhlawumbi kancinci [1]
- ☐ Ngamanye amaxesha [2]
- ☐ Phantse rhonqo [3]
- ☐ Rhoqo [4]

20.3

Unable to Control

Kule nyanga iphelileyo kungangaphi uziva ukuba awukwazanga ukulawula izinto ezibalulekileyo ebomini bakho.

Expects a single option response (**required**)

- ☐ Zange [0]
- ☐ Kancinci kakhulu [1]
- ☐ Ngamanye amaxesha [2]
- ☐ Kaninzana [3]
- ☐ Kaninzi kakhulu [4]

20.4

Nervous and Stressed

Kulenyanga idlulileyo kukangaphi uziva unovalo kwaye uphantsi koxinzelelo.

Expects a single option response (**required**)

- ☐ Zange [0]
- ☐ Kancinci kakhulu [1]
- ☐ Ngamanye amaxesha [2]
- ☐ Kaninzana [3]
- ☐ Kaninzi kakhulu [4]

20.5

Confident in Personal Problems

Kulenyanga idlulileyo kukangaphi usiva ukuzithemba ngokumelana neengxaki zakho?

Expects a single option response (**required**)

- Zange [0]
- Kambalwa kakhulu [1]
- Ngamanye amaxesha [2]
- Kaninzana [3]
- Kaninzi kakhulu [4]

20.6

Things Going Your Way

Kulenyanga idlulileyo kukangangakanani usiva ukuba izinto zihamba ngokweminqweno yakho?

Expects a single option response (**required**)

- Zange [0]
- Kancinci kakhulu [1]
- Ngamanye amaxesha [2]
- Kaninzana [3]
- Kaninzi kakhulu [4]

20.7

Not Coping

Kulenyanga idlulileyo kukangakanani usiva ukuba awukwazi ukumelana nazo zonke izinto omele kukuzenza?

Expects a single option response (**required**)

- Zange [0]
- Kancinci kakhulu [1]
- Ngamanye amaxesha [2]
- Kaninzana [3]
- Kaninzi kakhulu [4]

20.8

Controlling Irritations

Kule nyanga idlulileyo kukangaphi ukwazi ukumelana nezinto ezikuhluphayo ebomini bakho?

Expects a single option response (**required**)

- Zange [0]
- Kancinci kakhulu [1]
- Ngamanye amaxesha [2]
- Kaninzana [3]
- Phantse rhoqo [4]

20.9

On Top of Things

Kule nyanga idlulileyo kukangakanani usiva ukuba izinto zikuhambela kakuhle?

Expects a single option response (**required**)

- Zange [0]
- Kancinci kakhulu [1]
- Ngamanye amaxesha [2]
- Kaninzana [3]
- Kaninzi kakhulu [4]

20.10

Felt Anger

Kule nyanga idlulileyo kukangakanani usiba nomsindo kuba izinto bezingaphaya kwamandla akho?

Expects a single option response (**required**)

- Zange [0]
- Kancinci kakhulu [1]
- Ngamanye amaxesha [2]
- Kaninzana [3]
- Kaninzi kakhulu [4]

20.11

Overcoming Difficulties

Kule nyanga idlulileyo kungangaphi ifumanisa ukuba iingxaki ziyakongamela de awakwazi ukumelana nazo?

Expects a single option response (**required**)

- ☐ Zange [0]
- ☐ Kambalwa kakhulu [1]
- ☐ Ngamanye amaxesha [2]
- ☐ Kaninzana [3]
- ☐ Phantse rhoqo [4]
